# The characteristics of influenza-like illness management in Japan

**DOI:** 10.1186/s12889-020-08603-x

**Published:** 2020-04-28

**Authors:** Shinya Tsuzuki, Keisuke Yoshihara

**Affiliations:** 1 Department of Paediatric Infectious Diseases, Institute of Tropical Medicine, Nagasaki University, 1-12-4 Sakamoto, Nagasaki, 852-8523 Japan; 2 AMR Clinical Reference Center, Disease Control and Prevention Center, National Center for Global Health and Medicine, Tokyo, Japan; 3 Faculty of Medicine and Health Sciences, University of Antwerp, Antwerp, Belgium

**Keywords:** Influenza, Clinical practice, Healthcare-seeking behaviour, Disease burden, Rapid influenza diagnostic test

## Abstract

**Background:**

This study aimed to make a quantitative assessment of the management of influenza-like illnesses (ILI) in Japanese healthcare settings.

**Methods:**

We analysed participants’ healthcare-seeking behaviour and physicians’ practice in January 2019 using an online survey of 200 households in Japan. Quality of life score, quality-adjusted life years lost, the duration of symptoms, and the duration of absence from work were compared between the influenza ILI group and the non-influenza ILI group with one-to-one propensity score matching. Missing data were imputed using multiple imputation.

**Results:**

In total, 261 of the 600 (43.5%) participants had at least one episode of influenza-like illness during January 2019. Of these, 194 (75.5%) visited healthcare facilities, 167 (86.1%) within 2 days of onset of symptoms. A total of 169 out of 191 (88.5%) received a rapid influenza diagnostic test and 101 were diagnosed with influenza, of whom 95.0% were treated with antivirals. The median quality-adjusted life-years (QALYs) lost was 0.0055 (interquartile range, IQR 0.0040–0.0072) and median absence from work for a single episode of influenza-like illness was 2 days (IQR 1–5 days). Albeit QALYs lost per episode was not different between two groups, the influenza ILI group showed longer duration of absence from work (5 days, IQR 4–6 days) than the non-influenza ILI group (2 days, IQR 1–3 days).

**Conclusions:**

In Japan, most people with influenza-like illnesses visit healthcare facilities soon after symptoms first occur and receive a diagnostic test. Those with influenza are usually treated with antivirals. Absence from work was longer for influenza than other similar illnesses.

## Background

Seasonal influenza generally occurs in regular annual epidemics and its disease burden is substantial [1–3]. However, it is difficult to evaluate the disease burden precisely because clinical manifestations and severity of influenza infection vary considerably [4]. Most influenza cases are mild and self-limiting, or even asymptomatic [5]. It is therefore difficult to estimate the total number of people with the disease. The concept of influenza-like illness (ILI) adds further complexity, with other respiratory viruses such as rhinovirus and respiratory syncytial virus having similar symptoms to influenza [6–8]. Physicians in most countries do not use virological tests for ILI patients because many of the symptoms are mild and test results will not affect disease management. Most countries therefore use ILI surveillance as an approximate indicator of influenza levels [1, 2], even though this may both undercount actual influenza infections and include some other respiratory infections.

The concept of medically-attended influenza may be helpful in identifying the disease burden of influenza. The burden from seasonal influenza has two aspects. The first is severe disease and deaths, and the second is the economic impact from large number of mild cases which result in absence from work, losses to production, and costs to health and social care services [1]. Deaths will be covered in official statistics but it is difficult to evaluate the economic impact of medically-attended influenza because it depends on the national healthcare system, social norms, and physicians’ practice [9, 10].

Discussion with infectious disease physicians suggests that Japan has distinct practices for ambulatory care for ILI, but no previous studies have examined the characteristics of ILI management in Japan. We believe that Japanese ILI management has four characteristics that differ significantly from practice in EU/EEA and North American countries:

1. High proportion of medically-attended influenza among those with symptomatic ILI;
2. Patients make early visits to healthcare facilities;
3. Rapid influenza diagnostic test (RIDT) for most cases; and
4. Antivirals are provided for most diagnosed cases of influenza.

Previous studies from EU countries and the United States suggest that less than half of ILI patients with influenza-like illnesses visit healthcare facilities, and the proportion of medically-attended influenza is low in these countries [3, 11–15]. However, the situation may be different in Japan. To our knowledge, no previous study has focused on the timing of visits to healthcare facilities by ILI patients. Akaishi and colleagues [16] suggested that the median time between onset of influenza-like symptoms and visiting hospitals in Japan was 26.2 h. Fowlkes and colleagues, however, reported that 36.0% of medically-attended influenza cases had visited healthcare facilities more than 2 days after symptom onset [17]. It is difficult to compare these two results directly, but 26.2 h (about 1 day) from symptom onset seems very early.

The third issue is the popularity of RIDT in Japan. The concept of ILI is not popular in Japan and they prefer a diagnosis of “influenza” to “ILI” or “common cold”. Contrary to the recommendation of the Infectious Disease Society of America [18], Japanese physicians usually use RIDT, rather than a molecular assay such as reverse-transcription polymerase chain reaction (RT-PCR). This may be because the rapid test has extremely high sensitivity and specificity in the early phases of symptoms [16, 19–21] and most patients in Japan visit physicians in these early stages, making the rapid test more appropriate.

Finally, both patients and physicians in Japan prefer antiviral treatment. In the United States in 2009–2010, only 36% of patients clinically diagnosed with influenza were treated with antivirals [11] and only 20.4% of medically-attended influenza cases were prescribed antivirals between 2009 and 2016 [17]. In Japan, most patients request antivirals when they have a diagnosis of influenza and physicians do not hesitate to prescribe them although there is no publicly available data on the benefits.

The ILI management in Japan therefore has several distinct characteristics but quantitative data and available evidence are scarce. This study’s primary objective was to identify the characteristics of management of these diseases in Japan, looking at both patients’ healthcare-seeking behaviour and physicians’ clinical practice. We also evaluated the disease burden of both influenza cases diagnosed using RIDT and similar illnesses caused by other respiratory viruses.

## Methods

### Setting

We conducted an online survey of 600 people in 200 households. The participants were voluntarily and randomly recruited from registrants of NEO MARKETING INC, a Japanese marketing research company. The basic characteristics of registrants are shown in Table S1 in Appendix 1. The original version of the questionnaire created by the authors is also available as Appendix 2. The survey period was during February 2019 and participants were asked to answer about episodes of ILI which they or their family members had experienced during January 2019. We defined ILI as symptoms measured fever of ≥38 C° and cough, in accordance with the definition by World Health Organization [22]. Only one person per household could respond and that person answered question about the whole household. Responders had to be at least 18 years old. Informed consent was given before starting the survey. There was no monetary incentive to complete the questionnaire. The survey included questions about demographic data such as gender, age, number of family members, household income, education, past medical history, and smoking habits of family members. Where a respondent or family member had ILI symptoms during January 2019, the respondent answered questions about the duration of symptoms, healthcare-seeking behaviour (healthcare facility visit, days between symptom onset and healthcare facility visit, and vaccination status for seasonal influenza), and their physicians’ practice (RIDT use, prescription of antivirals, and class of antivirals prescribed).

### Statistical analysis

As described in “*Setting*”, we obtained data of 600 persons from 200 responders’ answer. We used these 600 persons’ data to conduct descriptive analysis about their basic characteristics and healthcare seeking behaviour. Besides descriptive analysis of online survey data, we estimated disease burden of influenza with 200 responders’ data by quality of life (QOL), quality-adjusted life-years (QALYs) lost, and duration of absence from work. Of those 200 responders, who were diagnosed as influenza when they have ILI symptoms by their physicians were classified as “influenza ILI group” and who were diagnosed as ILI caused by respiratory viruses other than influenza viruses (e.g. rhinovirus, respiratory syncytial virus, and so forth) were classified as “non-influenza ILI group”. We also compared these indicators between the two groups. Those who did not visit any healthcare facility while they have ILI symptoms were excluded from this comparison.

SF-12v2 Standard, Japanese questionnaire (SF-12v2® Health Survey© 1994, 2002, 2009 Quality Metric Incorporated, Medical Outcomes Trust and Shunichi Fukuhara. All rights reserved) [23] was included in the questionnaire to estimate the QOL at the onset of symptoms and QALYs lost by each episode of illness. In principle, responders answered SF-12v2 questionnaire for their own health status. They also answered about their children’s health status because the survey excluded respondents under 18 years old. QOL values were calculated using the method of Brazier and colleagues [24]. QALYs lost to each ILI episode was calculated as:

$$ QALYs\ lost=\left(1-\mathrm{QOL}\right)\times \frac{duration\ of\ symptoms\ (days)}{365} $$

The duration of absence was defined as the number of days in which patients or their caregivers had to take leave from work.

We compared the difference in QOL values, QALYs lost, duration of symptoms, and duration of absenteeism between the two groups using multiple imputation [25] to handle missing data. The imputation procedure uses all the known covariates thought to be associated with the missingness mechanism to help predict the values of missing items. A scales logit transformation was chosen to give normally distributed and plausible values. The results across 10 imputed datasets were combined using Rubin’s rules. For comparison, we also performed the analysis on the subset of complete cases.

We used linear regression analyses with one-to-one propensity score matching (one-to-one nearest neighbour pair matching, calliper = 0.2) [26] calculated by multivariate logistic regression model predicting the likelihood of diagnosis of influenza as opposed to other ILIs. We included age, sex, risk factor for severe illness, smoking, vaccination history for seasonal influenza, household income, education level, antibiotic prescription, and QOL value in the model to calculate propensity score. Two-sided *p*-values of < 0.05 were considered to show statistical significance. As a sensitivity analysis, we used inverse-probability weighted propensity score matching (IPW-PS) analysis instead of one-to-one matching. All statistical analyses used R, version 3.6.1 [27].

## Results

### Population characteristics and participants’ behaviour

In total, 261 of 600 (43.5%) participants had at least one episode of ILI influenza-like illness during January 2019. Of these, 194 (75.5%) visited healthcare facilities, 167 (86.1%) of those within 2 days of symptom onset. A total of 88.5% of these patients were tested using RIDT and 101 were diagnosed as having influenza, of whom 95.0% were given antivirals. The details of the descriptive analysis are shown in Table 1.

**Table 1:** Demographic and behavioural characteristics of the participants

Variable	Number (Percentage) or median (IQR)
Number of household members	4 (3–4)
Male	295/600 (49.2%)
Age (year)	42 (21–57)
High-risk group^a^	46/201 (22.9%)x
Smoker	81/600 (13.5%)
Healthcare facility visit	194/261 (75.5%)
Duration of symptoms (days)	2 (1–3)
Day of healthcare facility visit (days from symptom onset)	1 (0–2)
Patients examined by RIDT	169/191 (88.5%)
Patients diagnosed as influenza at healthcare facility	101/194 (52.1%)
Influenza diagnosed by RIDT	97/101 (96.0%)
Treated by antivirals among influenza cases diagnosed by RIDT	96/101 (95.0%)
Class of antivirals prescribed	
Oseltamivir	22 (37.3%)
Baloxavir	20 (33.9%)
Laninamivir	9 (15.3%)
Zanamivir	4 (6.8%)
Unknown	4 (6.8%)
Vaccinated for seasonal influenza	87 (34.5%)
Income level of household	
< 50,000 USD^b^/year	64 (32.0%)
50,000 USD/year < < 100,000 USD/year	86 (43.0%)
> 100,000 USD/year	28 (14.0%)
Education level of householder	
Primary	1 (0.5%)
Secondary	83 (46.5%)
Tertiary	95 (47.5%)
Advanced	11 (5.5%)

*IQR* Interquartile range, *RIDT* Rapid influenza diagnostic test, *USD* US dollars

^a^Participants who have past medical history associated with high-risk of severe influenza

^b^1 USD = 100 Japanese yen

### Disease burden

The median value of QOL and QALYs lost during the period of ILI were 0.67 (interquartile range [IQR] 0.60–0.79) and 0.0055 (IQR 0.0040–0.0072). The median duration of symptoms and absenteeism were 2 days (IQR 1–3 days) and 2 days (IQR 1–5 days).

### Difference between influenza and other ILIs

**Fig. 1:**
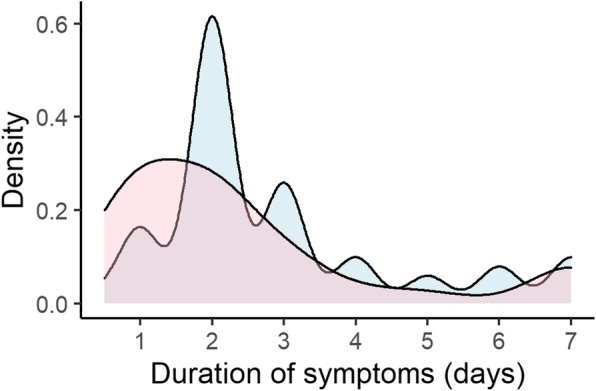
Probability density curve of duration of symptoms in the influenza ILI group and the non-influenza ILI group. ILI; influenza like illness. Blue area represents the influenza ILI group and Red area represents the non-influenza ILI group

**Fig. 2:**
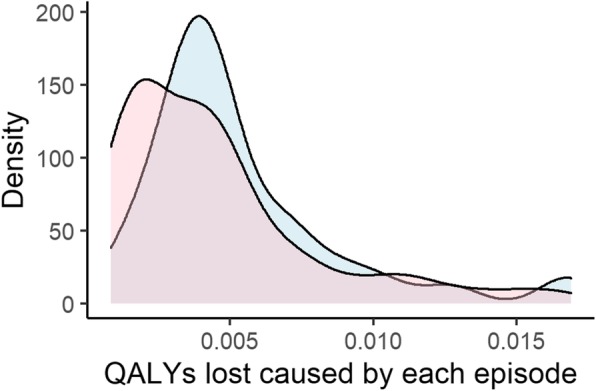
Probability density curve of QALYs lost in the influenza ILI group and the non-influenza ILI group. ILI; influenza like illness, QALYs; quality-adjusted life years Blue area represents the influenza ILI group and Red area represents the non-influenza ILI group

**Fig. 3:**
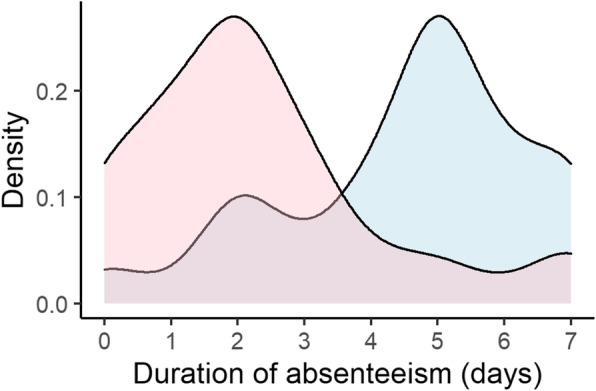
Probability density curve of duration of absenteeism in the influenza ILI group and the non-influenza ILI group. ILI; influenza like illness. Blue area represents the influenza ILI group and Red area represents the non-influenza ILI group

The median QOL score during symptomatic period of the influenza ILI group and the non-influenza ILI group was 0.66 (IQR 0.58–0.79) and 0.66 (IQR 0.59–0.79). The median QALYs lost per episode was 0.0044 (IQR 0.0034–0.0066) in the influenza ILI group and the non-influenza ILI group were 0.0044 (IQR, 0.0034–0.0066) and 0.0036 (IQR 0.0018–0.0054), respectively. The basic characteristics of the influenza ILI group and the non-influenza ILI group are shown in Table 2. Figure 1, Fig. 2, and Fig. 3 show the difference between two groups in duration of symptoms, QALYs lost per episode, and duration of absenteeism, respectively.

**Table 2:** Characteristics and disease burden of the influenza ILI group and the non-influenza ILI group

Variable	Influenza ILI group (***N*** = 72)	Non-influenza ILI group (***N*** = 73)
Number of household members	4 (2–4)	3 (2–4)
Male	34 (47.9%)	46 (63.8%)
Age	42 (17–53)	42 (22–55)
High-risk group	15 (20.8%)	22 (30.1%)
Smoker	10 (13.9%)	19 (26.0%)
Day of healthcare facility visit (days from symptom onset)	1 (1–2)	1 (1–2)
Patients examined by RIDT	71 (97.2%)	57 (80.3%)
Treated by antivirals	38 (90.5%)	1 (4.3%)
Vaccinated for seasonal influenza	28 (38.9%)	22 (31.9%)
Income level		
< 50,000 USD^a^/year	20 (30.3%)	22 (33.8%)
50,000 USD/year < < 100,000 USD/year	34 (51.5%)	34 (52.3%)
> 100,000 USD/year	12 (18.2%)	9 (13.8%)
Education level of householder		
Primary	0	0
Secondary	33 (45.8%)	32 (43.8%)
Tertiary	37 (51.4%)	34 (46.6%)
Advanced	2 (2.8%)	7 (9.6%)
Duration of symptoms (days)	2 (2–3)	2 (1–3)
QOL during symptomatic period	0.66 (0.58–0.79)	0.66 (0.59–0.79)
QALYs lost per episode	0.0044 (0.0034–0.0066)	0.0036 (0.0018–0.0054)
Duration of absenteeism (days)	5 (4–6)	2 (1–3)

Values are shown as absolute number (percentage) or median (interquartile range)

*RIDT* Rapid influenza diagnostic test, *QOL* quality of life, *QALYs* quality-adjusted life-years, *USD* US dollars

^a^1 USD = 100 Japanese Yen

In addition, we compared outcomes (QOL score, QALYs lost, duration of symptoms and duration of absenteeism) of vaccinated group and unvaccinated group in order to examine the influence of vaccination for seasonal influenza on the course of illness. We found no significant difference between two groups and details of the results are shown in Table 3.

**Table 3:** Differences in outcomes between vaccinated group and unvaccinated group

Outcome	Vaccinated^**b**^	Unvaccinated^**b**^	***p***-value
QOL score^a^			
Influenza ILI group	0.657 (0.585–0.765)	0.660 (0.586–0.795)	0.668
Non-influenza ILI group	0.677 (0.632–0.723)	0.660 (0.580–0.817)	0.694
QALYs lost^a^			
Influenza ILI group	0.00427 (0.00360–0.00529)	0.00456 (0.00329–0.00684)	0.791
Non-influenza ILI group	0.00362 (0.00232–0.00456)	0.00379 (0.00169–0.00506)	0.824
Duration of symptoms^b^			
Influenza ILI group	2.0 (2.0–3.0)	2.0 (2.0–3.5)	0.834
Non-influenza ILI group	2.0 (1.0–3.0)	2.0 (1.0–3.0)	0.832
Duration of absenteeism^b^			
Influenza ILI group	5.0 (2.5–5.5)	5.0 (4.0–6.0)	0.513
Non-influenza ILI group	2.0 (1.0–3.0)	2.0 (1.0–3.0)	0.944

*ILI* influenza like illness, *QOL* quality of life, *QALYs* quality-adjusted life-years

^a^These outcomes were compared by Mann-Whitney *U* test

^b^ Values are shown as median (interquartile range)

We imputed the missing values in the dataset before comparing the two groups. The number of missing items is shown in Table S2 in the Appendix 1. After one-to-one propensity score matching for the influenza ILI group versus the non-influenza ILI group with imputed data, differences in QOL score, QALYs lost, and duration of symptoms were not statistically significant. However, those with influenza were off work about 2 days longer than the other group. The comparison is shown in Table 4.

**Table 4:** Differences in outcomes between the two group after propensity score matching

Outcome	Estimate	SE	***p***-value
QOL score			
Intercept	0.698	0.0174	< 0.001
Influenza ILI group	−0.0231	0.0228	0.314
QALYs lost			
Intercept	0.00413	0.000503	< 0.001
Influenza ILI group	0.000650	0.000620	0.297
Duration of symptoms			
Intercept	2.189	0.250	< 0.001
Influenza ILI group	0.456	0.346	0.192
ss			
Intercept	2.099	0.277	< 0.001
Influenza ILI group	2.010	0.392	< 0.001

*ILI* influenza like illness, *QOL* quality of life, *QALYs* quality-adjusted life-years,

*SE* standard error

### Sensitivity analysis

We used one-to-one propensity score matching for the influenza group versus the other group for responses with complete data only. We also used IPW-PS analysis with the imputed dataset as a sensitivy analysis.

Only 58 of the 200 cases contained complete data. After one-to-one propensity score matching, we had 13 pairs of cases, providing 26 complete cases for analysis. Linear regression analysis showed that duration of absenteeism was significantly different between the two groups, but there were no significant differences between QOL score, QALYs lost, and duration of symptoms. The details are shown in Table S3 in the Appendix 1.

IPW-PS analysis with the imputed dataset showed that QOL scores were similar between the two groups, but duration of symptoms and absence were longer for those with influenza ILI. As a result, QALYs lost was also greater for those with influenza ILI. These results are shown in Table S4 in the Appendix 1.

## Discussion

The result of this study suggests that most ILI patients in Japan visited healthcare facilities soon after the onset of symptoms and most physicians used RIDT to assess them. Most people with a diagnosis of influenza were given antivirals. These preferences were observed in our study and are also consistent with the expectations of experts. However, they are quite different from ILI management in other countries [3, 17]. Direct medical costs from medically-attended influenza can be considered one of the main parts of the influenza disease burden [28, 29], so the high proportion of medically-attended influenza in Japan contributes to a heavier disease burden than in EU/EEA countries and the United States.

Our analysis showed that QOL score during the symptomatic period did not differ between those with influenza ILI and non-influenza ILIs. However, this should be interpreted carefully because most people with influenza received antivirals, which might also reduce the severity of ILI [30]. If antiviral treatment had not been provided, the QOL score of people with influenza ILI might have been worse than people with other non-influenza ILIs. The effect of antiviral treatment also modifies the duration of symptoms [31], so without antivirals, the duration of influenza ILI symptoms might be longer than non-influenza ILIs. The IPW-PS analysis, however, showed different results in the sensitivity analysis. Previous studies reported differently about difference of QOL score and duration of symptoms between influenza ILI and non-influenza ILIs [32, 33], so further work would be helpful in this specific area.

The duration of absence varied between the two groups in spite of the similar duration of symptoms. This may be because the Japanese School Health and Safety Act [34] provides that school-age children with influenza have to remain at home for 5 days after symptom onset, but does not define the duration of absence for other non-influenza ILIs. Adults with school-age children therefore often have to take nursing leave even after the children’s recovery from symptoms. This regulation might increase the societal burden of influenza [35]. Asymptomatic influenza patients have weaker infectivity than those with symptoms [4], so it is possible that a five-day absence is longer than necessary.

One strength of this study is that the methodology enabled us to maximize the amount of information from the original data. Our data included only 200 households, which might be a limitation. However, the multiple imputation process allowed us to include all respondents’ answers in our analysis and propensity score matching ensured the robustness of our comparison. Another strength is that our results identified the distinct characteristics of Japanese ILI management in ambulatory care settings. To our knowledge, this is the first study to focus quantitatively on the proportion of medically-attended influenza, antiviral treatment, and days between symptom onset and healthcare facility visit. These findings will help to estimate the national disease burden of ILI and could provide a baseline for future studies.

The study had several limitations. First, the SF-12v2 questionnaire is not the standard tool for calculating QOL scores in Japan. Some researchers have tried to estimate QOL scores using SF-12v2 in Japan, drawing on Brazier and colleagues [24], but this method has not yet been officially accredited.

Second, we could not stratify the influenza ILI group into “treated” and “untreated” because almost all of them were given antivirals. Comparison between those with influenza ILI and other non-influenza ILIs might be a good proxy, but ideally comparison among those with the same diagnosis would be better.

Third, our data were based on an online survey. Unlike conventional questionnaire surveys, online surveys require participants to have basic internet literacy. The data may therefore not be fully representative. Additionally, we have no data about their response rate to the questionnaire. It is possible that the response rate was low so that only people deeply interested in ILI answered the questionnaire then the data we obtained were biased. For example, the proportion of vaccinated participants in the influenza ILI group was higher than that of in the non-influenza ILI group. It seems paradoxical because vaccinated people usually show lower risk of influenza infection. This can be explained by higher interest in their health status and their healthcare seeking behaviour in the vaccinated group because diagnosis of influenza is made by physicians. Considering this, participants of our survey might have higher interest in their health status than Japanese general population do, especially in the influenza ILI group.

Forth, the comparison about duration of symptoms showed different results between one-to-one propensity score matching and IPW-PS analysis. In the present study, both methods have their own merits and challenges. In one-to-one matching, we have to discard some cases due to mismatch although we can avoid extreme weighting for each case. Our data do not have a large number of participants then we would like to keep all cases, if possible. Conversely, if we use IPW-PS, we can use all cases in our analysis, however, some cases might be extremely weighted. We adopted one-to-one matching as the main method in accordance with the principle of propensity score method [26] in spite of its limitation. Then therefore we believe that IPW-PS is an appropriate option for sensitivity analysis. The results were partially inconsistent between these two methods, then we are not sure whether duration of symptoms is different between influenza and ILI or not. Last, our survey results are based on participants’ self-reported answers. It is possible that some participants do not understand what their physicians told them then therefore some of their answers might be inaccurate.

## Conclusions

This study showed that ILI management in Japan has distinct characteristics. ILI patients tend to visit healthcare facilities soon after onset of symptoms, and physicians use RIDT to detect influenza, and then prescribe antivirals in most cases. These behaviours and practices might influence the disease burden of ILI in Japan, and further work to evaluate the situation more fully would be helpful.

## Supplementary information

**Additional file1.** The Characteristics of Influenza-Like Illness Management in Japan.

**Additional file 2.** Questionnaire used to obtain data used for the present study (English translation).

## Data Availability

All data used in the manuscript are derived from the results of questionnaire survey conducted by a private company. The raw data are available upon reasonable request to the corresponding author. The authors have access to the raw data without obtaining any special permissions from the company because the raw data do not include any personal information can be connected to each participant.

## Abbreviations

EU/EEA: European Union/European Economic Area
ILI: Influenza like illness
IPW-PS: Inverse-probability weighted propensity score matching
IQR: Interquartile range
QALYs: Quality-adjusted life years
QOL: Quality of Life
RIDT: Rapid influenza diagnostic test
RT-PCR: Reverse-transcription polymerase chain reaction
SE: Standard error
USD: United States Dollar
