# Supplementary material for: The characteristics of influenza-like illness management in Japan
Source: BMC Public Health. 2020 Apr 28;20:568. doi: 10.1186/s12889-020-08603-x (PMC7189553; doi:10.1186/s12889-020-08603-x)
Supplement: Supplementary file 1 — Additional file1. The Characteristics of Influenza-Like Illness Management in Japan. [file 12889_2020_8603_MOESM1_ESM.docx]

**Appendix 1**

**The Characteristics of Influenza-Like Illness Management in Japan**

Shinya Tsuzuki^1,2,3*^ and Keisuke Yoshihara^1^

1 Department of Paediatric Infectious Diseases, Institute of Tropical Medicine, Nagasaki University, Nagasaki, Japan

2 Disease Control and Prevention Center, National Center for Global Health and Medicine, Tokyo, Japan

3 Faculty of Medicine and Health Sciences, University of Antwerp, Antwerp, Belgium

**Contents**

**Table S1. Basic characteristics of people registered with NEO MARKETING INC.**

**Table S2. Number of missing values in the original data**

**Table S3. Difference in outcomes between the two groups after propensity score matching with complete data only**

**Table S4.** **Difference in outcomes between the two groups after inverse probability-weighted propensity score analysis**

**Table S1. Basic characteristics of people registered with NEO MARKETING INC.**

| **Variable** | **Number (Percentage) or median (IQR)** | | |
| --- | --- | --- | --- |
| **Male** | **52.9%** | | |
| **Age (year)** | **<30** | **9.2%** | |
|  | **30s** | **17.4%** | |
|  | **40s** | **26.2%** | |
|  | **50s** | **25.0%** | |
|  | **60s** | **16.0%** | |
|  | **>70** | **6.2%** | |
| **Marital status** | **Married** | **51.4%** | |
|  | **Unmarried** | **43.3%** | |
|  | **Widowed** | **5.3%** | |
| **Having children** | **41.7%** | | |
| **Income level of household** | **< 50,000 USD/year** | | **56.7%** |
|  | **50,000 USD/year <**  **< 100,000 USD/year** | | **25.4%** |
|  | **> 100,000 USD/year** | | **10.5%** |
|  | **Unknown** | | **7.4%** |

USD: US dollars, 1 USD = 110 Japanese yen**Table S2. Number of missing values in the original data**

| **Variable** | **Number of missing values (%)** |
| --- | --- |
| **Number of household members** | **0** |
| **Sex** | **3 (1.5%)** |
| **Age** | **0** |
| **High-risk group** | **0** |
| **Smoker** | **0** |
| **Day of healthcare facility visit**  **(days from symptom onset)** | **5 (2.5%)** |
| **Patients examined by RIDT** | **8 (4.0%)** |
| **Treated by antivirals** | **86 (43.0%)** |
| **Vaccinated for seasonal influenza** | **7 (3.5%)** |
| **Income level** | **22 (11.0%)** |
| **Education level of householder** | **0** |
| **Duration of symptoms (days)** | **0** |
| **QOL during symptomatic period** | **0** |
| **QALYs lost per episode** | **0** |
| **Duration of absenteeism (days)** | **0** |

QOL: quality of life, QALYs: quality-adjusted life-years, RIDT: rapid influenza diagnostic test

**Table S3. Difference in outcomes between the two groups after propensity score matching with complete data only**

| **Outcome** | **Estimate** | **SE** | ***p*-value** |
| --- | --- | --- | --- |
| **QOL score**  **Intercept**  **Influenza group** | **0.666**  **0.0443** | **0.0346**  **0.0489** | **< 0.001**  **0.374** |
| **QALYs lost**  **Intercept**  **Influenza group** | **0.00567**  **0.000419** | **0.00127**  **0.00179** | **< 0.001**  **0.817** |
| **Duration of symptoms**  **Intercept**  **Influenza group** | **2.731**  **-0.192** | **0.548**  **0.774** | **< 0.001**  **0.806** |
| **Duration of absenteeism**  **Intercept**  **Influenza group** | **2.692**  **2.077** | **0.502**  **0.710** | **< 0.001**  **0.00739** |

QOL: quality of life, QALYs: quality-adjusted life-years, SE: standard error

**Table S4.** **Differences in outcomes between the two groups after inverse probability-weighted propensity score analysis**

| **Outcome** | **Estimate** | **SE** | ***p*-value** |
| --- | --- | --- | --- |
| **QOL score**  **Intercept**  **Influenza group** | **0.688**  **-0.0108** | **0.0134**  **0.0192** | **< 0.001**  **0.574** |
| **QALYs lost**  **Intercept**  **Influenza group** | **0.00392**  **0.00123** | **0.000360**  **0.000493** | **< 0.001**  **0.0140** |
| **Duration of symptoms**  **Intercept**  **Influenza group** | **2.156**  **0.600** | **0.196**  **0.263** | **< 0.001**  **0.0248** |
| **Duration of absenteeism**  **Intercept**  **Influenza group** | **2.112**  **2.382** | **0.197**  **0.339** | **< 0.001**  **< 0.001** |

QOL: quality of life, QALYs: quality-adjusted life-ears, SE: standard error
