# Supplementary material for: The characteristics of influenza-like illness management in Japan
Source: BMC Public Health. 2020 Apr 28;20:568. doi: 10.1186/s12889-020-08603-x (PMC7189553; doi:10.1186/s12889-020-08603-x)
Supplement: Supplementary file 2 — Additional file 2. Questionnaire used to obtain data used for the present study (English translation). [file 12889_2020_8603_MOESM2_ESM.docx]

**Appendix 2**

**Questionnaire used to obtain data for the present study**

This is a translation of the original Japanese questionnaire used for the survey. Responders answer all questions through an online system.

- Please answer your sex
- Please answer your age
- Please answer prefecture you live in
- How many people does your family have?
- Please answer the relationship between you and each your family member.
- Within a month, did you or your family member(s) have symptoms such as fever >38℃ and cough? Please specify the family member(s) who had the symptoms.
- Please answer the age of your family member(s).
- Does the person who had fever and cough have any past history shown below?
  - Asthma
  - Allergic rhinitis
  - Atopic dermatitis
  - Neurologic disease (Developmental retardation, cerebral paralysis, etc.)
  - Chronic lung diseases (COPD, etc.)
  - Cardiac diseases (congenital heart diseases, heart failure, myocardial infarction, angina, etc.)
  - Diabetes
  - Renal diseases (diabetic nephropathy, chronic glomerulonephritis, etc.)
  - Liver diseases (chronic hepatitis, etc.)
  - Metabolic diseases (Phenylketonuria, methylmalonic acidemia, etc.)
  - Immunodeficiency (HIV/AIDS, malignant tumour [cancer], etc.)
  - Pregnancy (when you had fever and cough)
  - Other diseases
- Please answer the person who smokes in your family (if there is).
- How long did your and/or your family member’s symptoms continue?
- How do you think about the source of infection which caused your and/or your family member’s symptoms?
  - Someone in school
  - Someone in workplace
  - Someone in family
  - Public place where people gather (parks, event sites, etc.)
  - Others
  - Do not know
- When you and/or your family member(s) had fever and symptoms, do you and/or your family member(s) visit any healthcare facility?
- If no, do you and/or your family member(s) use any drug during having symptoms?
- If you and/or your family member(s) use any drug, please answer the name of the drug.
- If you and/or your family member(s) visited a health facility, what kind of facility?
  - Primary care physician
  - Public general hospital
  - Private general hospital
  - University hospital or national center hospital
  - Do not know
- What was the diagnosis of you and/or your family member(s)?
  - Common cold
  - Bronchitis
  - Pneumonia
  - Otitis media
  - Influenza A
  - Influenza B
  - RS virus infection
  - Adenovirus infection
  - Group A streptococcus infection
  - Mycoplasma infection
  - Pertussis
  - Others (please specify)
- Please answer the name of drug prescribed when you and/or your family member(s) visited a healthcare facility.
- When did you and/or your family member(s) visited a healthcare facility after symptoms occurred?
- When you and/or your family member(s) visited a healthcare facility, were you and/or your family member(s) examined by influenza diagnostic test?
- Did you and/or your family member(s) admit due to these symptoms?
- How long did you and/or your family member(s) stay in the hospital? What was the diagnosis?
- When visited a healthcare facility, how much was the transportation cost?
- When visited a healthcare facility, how much did you and/or your family member(s) pay to the facility?
- When visited a healthcare facility, how much did you and/or your family member(s) pay to the pharmacy?
- How many days did you and/or your family member(s) take sick leave due to the symptoms?
- How many days did you and/or your family member(s) take nursing care leave due to other family member’s symptoms?
- Did you and/or your family member(s) who had the symptoms take influenza vaccine within six months?
- If your family member(s) is/are under 13 years old, did he/she take second dose of influenza vaccine?
- How much did the vaccine cost?
- When did you and/or your family member(s) take vaccine?

**[Next, the questionnaire included SF-12v2 Standard, Japanese questionnaire (SF-12v2® Health Survey © 1994, 2002, 2009 Quality Metric Incorporated, Medical Outcomes Trust and Shunichi Fukuhara. All rights reserved). Permission is required to access the original version]**

- Please answer your household income.
- Please answer your and your partner’s profession.
- Please answer your education level.
- Please answer your postcode.
